# Immunohistochemistry with Anti-BRAF V600E (VE1) Mouse Monoclonal Antibody is a Sensitive Method for Detection of the BRAF V600E Mutation in Colon Cancer: Evaluation of 120 Cases with and without KRAS Mutation and Literature Review

**DOI:** 10.1007/s12253-017-0344-x

**Published:** 2017-11-10

**Authors:** Katerina Dvorak, Amanda Higgins, John Palting, Michael Cohen, Patrick Brunhoeber

**Affiliations:** Roche Tissue Diagnostics, 1910 E. Innovation Park Drive, Tucson, AZ USA

**Keywords:** BRAF V600E, KRAS, Colon cancer, DNA sequencing, Immunohistochemistry

## Abstract

The major aim of this study was to evaluate the performance of anti-BRAF V600E (VE1) antibody in colorectal tumors with and without *KRAS* mutation. *KRAS* and *BRAF* are two major oncogenic drivers of colorectal cancer (CRC) that have been frequently described as mutually exclusive, thus the *BRAF* V600E mutation is not expected to be present in the cases with KRAS mutation. In addition, a review of 25 studies comparing immunohistochemistry (IHC) using the anti-BRAF V600E (VE1) antibody with BRAF V600E molecular testing in 4041 patient samples was included.

One-hundred and twenty cases with/without *KRAS* or *BRAF* mutations were acquired. The tissue were immunostained with anti-BRAF V600E (VE1) antibody with OptiView DAB IHC detection kit. The *KRAS* mutated cases with equivocal immunostaining were further evaluated by Sanger sequencing for *BRAF* V600E mutation. Thirty cases with *BRAF* V600E mutation showed unequivocal, diffuse, uniform, positive cytoplasmic staining and 30 cases with wild-type *KRAS* and *BRAF* showed negative staining with anti-BRAF V600E (VE1) antibody. Out of 60 cases with *KRAS* mutation, 56 cases (93.3%) were negative for *BRAF* V600E mutation by IHC. Four cases showed weak, equivocal, heterogeneous, cytoplasmic staining along with nuclear staining in 25–90% of tumor cells. These cases were confirmed to be negative for *BRAF* V600E mutation by Sanger sequencing. Overall, IHC with anti-BRAF V600E (VE1) antibody using recommended protocol with OptiView detection is optimal for detection of *BRAF* V600E mutation in CRC. Our data are consistent with previous reports indicating that *KRAS* and *BRAF* V600E mutation are mutually exclusive.

## Introduction

Colorectal cancer is the third most common cancer and the fourth most prevalent cause of death in the world [46]. Approximately 35–45% of patients with colorectal tumors have mutation in *KRAS* gene, while *BRAF* V600E mutation is found in about 5–15% of colorectal adenocarcinomas [8, 9, 26, 31]. Both these mutations are considered to be oncogenic driver mutations, since they are both responsible for the initiation and maintenance of the tumor [10]. Importantly, many studies have indicated that *BRAF* V600E mutation occurs only in tumors that do not carry mutations in *KRAS* gene and it is widely accepted that these two mutations are mutually exclusive [10, 23, 25, 30].

The *BRAF* gene encodes a cytoplasmic serine-threonine kinase that is frequently mutated in various cancers, including melanoma, papillary thyroid carcinoma, and colorectal carcinoma, among others. The oncogenic mutations in *BRAF* gene result in constitutive activation of the MAPK signaling pathway, leading to increased cell proliferation, resistance to apoptosis and tumor progression. The most common of these mutations, the V600E mutation, occurs in exon 15 and results in a substitution from valine to glutamic acid at position 600 within the BRAF kinase domain.

*BRAF* V600E mutation occurs in about 5% of microsatellite stable (MSS) CRC tumors. These tumors are associated with a distinct molecular and clinical phenotype with a poor prognosis [40]. The presence of *BRAF* V600E mutation in CRC is associated with poor survival [44]. *BRAF* V600E mutation is also detected in sporadic CRC tumors with microsatellite instability (MSI) [27]. Particularly, *BRAF* V600E mutation is observed in about two thirds of MSI tumors with the loss of MLH1 expression due to MLH1 promoter methylation [18]. In contrast, *BRAF* V600E mutation is very rare in CRC patients with Lynch syndrome [27]. In clinical practice it is much easier to detect *BRAF* V600E mutation than methylation status of MLH1 promoter [26]. Therefore, it was suggested that assessment of *BRAF* V600E mutation can be used to triage patients for mismatch repair (MMR) genetic testing to differentiate MLH1-deficient sporadic CRC from Lynch syndrome caused by germ-line *MLH1* mutations [7, 14, 17, 26, 41, 43]. Currently, the American National Comprehensive Cancer Network (NCCN) guidelines recommend that *BRAF* V600E mutational status should be evaluated in all colorectal carcinomas to identify 1) the patients with Lynch syndrome in MMR deficient group and 2) to identify the MMR proficient/BRAF V600E group with poor prognosis [15, 38, 43].

The most common approach for the detection of *BRAF* mutation is sequencing of tumor DNA, for example Sanger sequencing, pyrosequencing and high resolution melting. All of these methods are able to detect a mutant allele in a background of 5–20 fold excess of wild-type alleles. In contrast, immunohistochemistry allows direct visualization of the mutant protein in the tumor cells in tissue context. The anti-BRAF V600E (VE1) antibody is currently used to evaluate the *BRAF* V600E mutation status in various cancers including CRC [32]. This antibody is a mutation-specific mouse monoclonal antibody that was raised against a synthetic peptide representing the *BRAF* V600E mutated amino acid sequence from amino acids 596 to 606 (GLATEKSRWSG) [5, 6].

The primary goal of this study was to compare the performance of the anti-BRAF V600E (VE1) antibody to detect *BRAF* V600E mutation by IHC in colon cancer cases with/without *KRAS* mutation. Since concomitant *KRAS* and *BRAF* tumor mutations are considered mutually exclusive we wanted to confirm that the CRC cases carrying *KRAS* mutation show negative BRAF V600E staining by IHC with anti-BRAF V600E (VE1) antibody. In addition, we performed a review of 25 studies that compared IHC using anti-BRAF V600E (VE1) antibody with molecular testing for *BRAF* V600E mutation.

## Materials and Methods

### Tumor Specimens

A total of 120 formalin-fixed paraffin embedded (FFPE) tissues from patients with colorectal cancer were ordered from Avaden Biosciences and GLAS/Consultants in Human Biologics. The requested cases included 60 CRC cases with confirmed *KRAS* mutation, 30 CRC cases with confirmed *BRAF* V600E mutation and 30 CRC cases confirmed to be wild-type BRAF and wild-type KRAS. The presence/absence of these mutations was confirmed by molecular testing by the vendor.

### BRAF V600E Immunohistochemistry

Four 4 μm thick sections were cut from the FFPE blocks. The testing was performed using anti-BRAF V600E (VE1) mouse monoclonal primary antibody (Ventana Medical Systems, Inc., Cat. Number 790–4855) the BenchMark ULTRA platform with Cell Conditioning 1 for 64 min, pre-peroxidase inhibition and primary antibody incubation for 16 min at 37 °C. Final concentration of the antibody was ~12 μg/ml. The OptiView DAB IHC Detection Kit (Ventana Medical Systems, Inc.) was used to detect *BRAF* V600E protein expression. Tissues were counterstained with Hematoxylin II (Ventana Medical Systems, Inc.) and Bluing Reagent (Ventana Medical Systems, Inc.) for 4 min. To measure the level of non-specific background signal, each tissue was also stained with a mouse monoclonal antibody (MOPC-21) [Negative Control (Monoclonal), Ventana Medical Systems, Inc.]. This antibody is not directed against any known epitope present in human tissue. In addition, slides containing 2 cases positive for *BRAF* V600E mutation (CRC, thyroid papillary carcinoma) and one case negative for *BRAF* V600E mutation (CRC) were used as run control slides. The absence/presence of the *BRAF* V600E mutation in the tissues was confirmed by Sanger sequencing. These slides were included with each individual run to assess the expected quality of the antibody and all components of the assay. The overall run was accepted if: 1) the *BRAF* V600E positive tissue control stained with anti-BRAF V600E (VE1) antibody showed specific cytoplasmic staining pattern and had acceptable background; 2) the positive tissue control stained with Negative Control Monoclonal shows no specific staining and had acceptable background; 3) the BRAF V600E negative tissue control stained with anti-BRAF V600E (VE1) antibody showed no specific staining pattern and acceptable background; and 4) the BRAF V600E negative tissue control stained with Negative Control (Monoclonal) shows no specific staining and has acceptable background.

The stain intensity of anti-BRAF V600E (VE1) antibody in tumor cells was recorded on a 0–3 scale. Strong cytoplasmic staining was scored as 3, medium cytoplasmic staining as 2, weak cytoplasmic staining as 1 and the absence of staining was scored as 0. In addition, any nuclear staining and the percentage of tumor cells stained positive with anti-BRAF V600E (VE1) antibody was recorded. The criteria for positive BRAF V600E staining included unequivocal, diffuse, uniform, cytoplasmic staining at intensity ≥1 in majority of malignant cells. The cases were scored as negative for BRAF V600E mutation if they showed no staining or weak, cytoplasmic, non-granular, uniform staining (stain intensity <1). The cases with staining of isolated tumor cells in a tumor that otherwise showed no staining were also scored as negative. The cases were scored as equivocal if they displayed ambiguous, heterogeneous, non-uniform cytoplasmic staining in tumor cells with or without nuclear staining. The equivocal cases were sequenced by Sanger sequencing to confirm the presence/absence of *BRAF* V600E mutation.

### BRAF V600E Sanger Sequencing

Genomic DNA was extracted from 20 μm thick sections from FFPE samples using the QIAamp FFPE Tissue Kit (Qiagen, Redwood, CA) according to manufacturer’s instructions. The primers for Sanger sequencing were designed to amplify region of the exon 15 of the *BRAF* gene coding sequences at mutation site and a few nucleotides in the intron on both ends. Two primers were used including 1) BRAF-ex15F-TGCTTGCTCTGATAGGAAAATG and 2) BRAF-ex15R-AGCATCTCAGGGCCAAAAAT. Both forward and reverse strands were sequenced on an Applied Biosystem’s 3730xl DNA Analyzer and analyzed using DNASTAR Lasergene 12 software (DNASTAR, Madison, WI).

## Results

### Anti-BRAF V600E (VE1) Immunohistochemistry

All 120 cases were examined for presence of *BRAF* V600E mutation by IHC using the anti-BRAF V600E (VE1) antibody on the automated VENTANA BenchMark ULTRA platform.

**Fig. 1:**
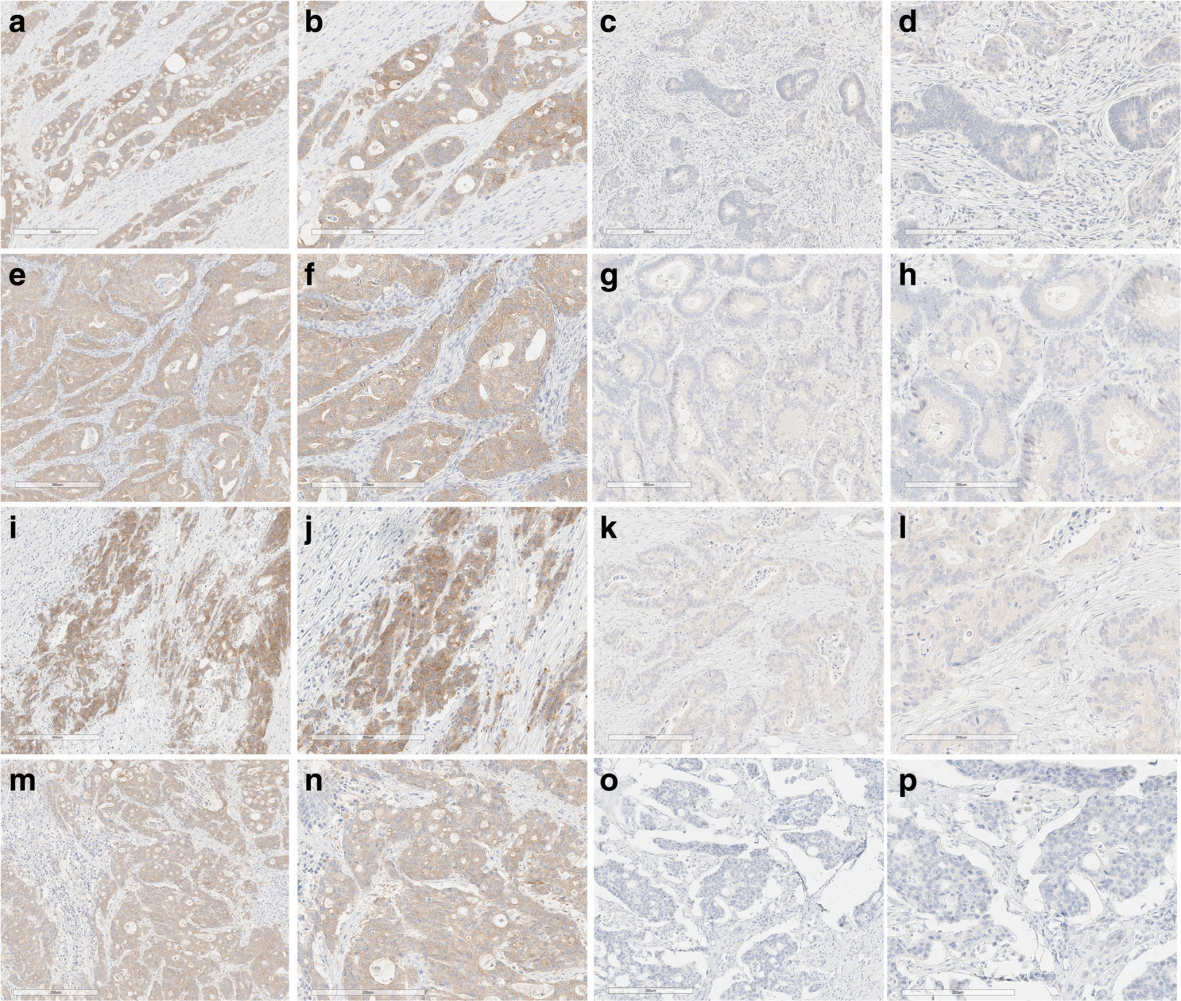
Representative images of eight colon cancer cases stained with anti-BRAF V600E (VE1) mouse monoclonal antibody. BRAF V600E mutation was confirmed in cases shown in images A,B,E,F,I,J,M,N by molecular testing, no BRAF V600E mutation was detected by molecular testing in cases shown in images C,D,G,H,K,L,O,P. Magnification 10× (A,C,D,E,G,I,K,M,O) and 20× (B,D,F,H,J,L,N,P)

All 30 cases with *BRAF* V600E mutation exhibited uniform, unequivocal, diffuse, cytoplasmic staining in majority of tumor cells with stain intensities of 1–2.75 and background ≤0.25. Out of 30 cases, 28 cases showed positive BRAF V600E signal in 100% of tumor cells and 2 cases showed positive staining in 90% and 85% of tumor cells, respectively. These data are consistent with previous reports indicating that majority of tumor cells express mutated *BRAF* V600E protein, since this mutation is driving tumor proliferation. All 30 cases with no *BRAF* V600E and no *KRAS* mutations showed intensities of ≤0.5 and background ≤0.25. In 28 cases the stain intensities were 0–0.25, in remaining two cases 10% and 85% of malignant cells stained at the stain intensities 0.5. The sensitivity and specificity was 100% for cases with confirmed *BRAF* V600E mutational status. Representative images are shown in Fig. 1.

**Fig. 2:**
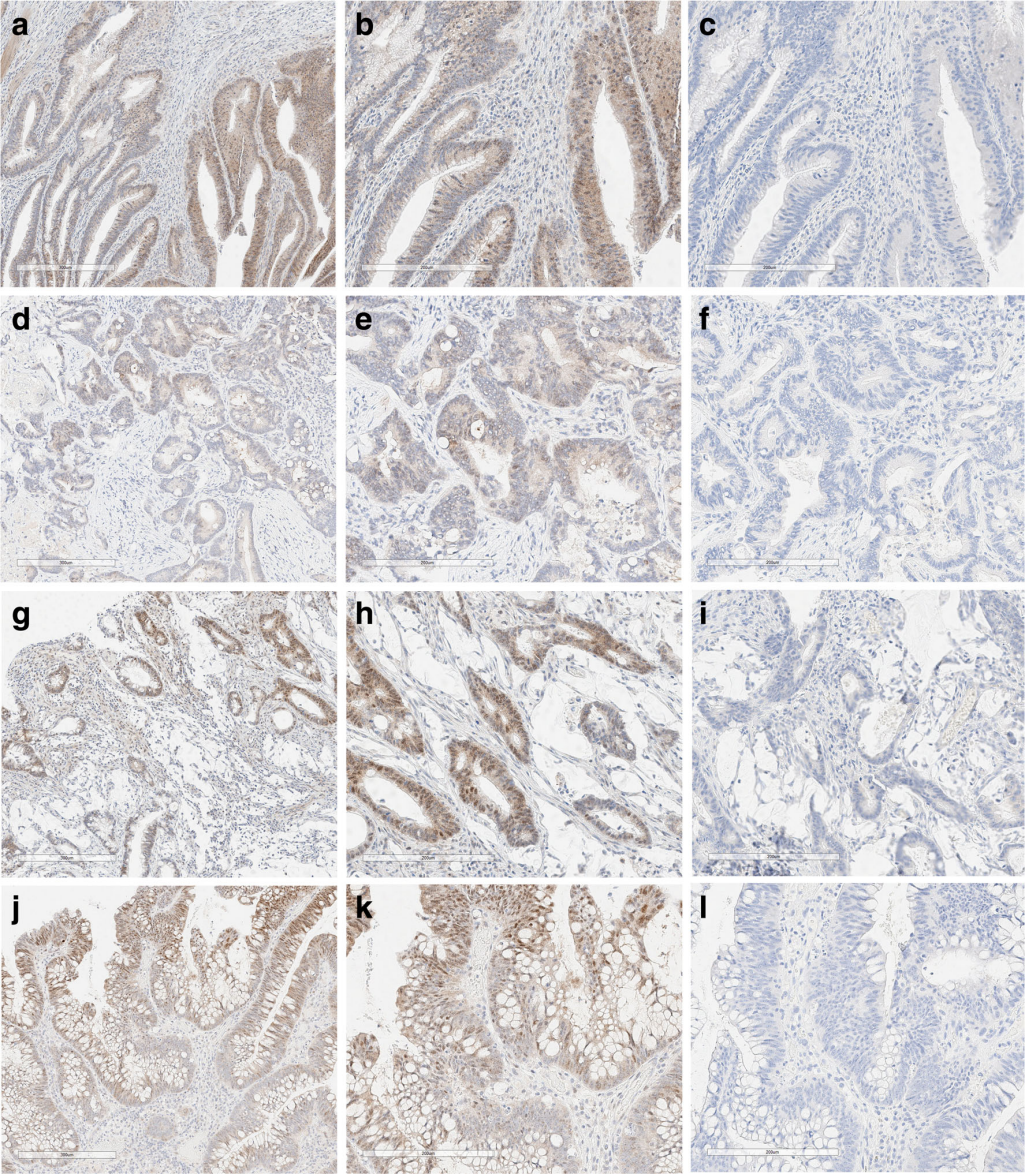
Representative images of four colon cancer cases with KRAS mutation showing equivocal staining. The tissues were stained with anti-BRAF V600E (VE1) mouse monoclonal antibody (A,B,D,E,G,H,J,K) and negative reagent control (C,F,I,L) [A,B,C - case 1; D,E,F - case 2; G,H,I - case 3; J,K,L - case 4, Magnification - 10× (A,D,G,J), 20× (B,C,E,F,H,I,K,L)]

Out of 60 CRC cases with *KRAS* mutations 56 cases were scored as negative for *BRAF* V600E mutation (stain intensity <1). There were four cases where the stain intensities were scored as 1. However, these four cases exhibited ambiguous, heterogeneous, non-uniform cytoplasmic staining along with nuclear staining and thus they were scored as equivocal. In the first case, only 40–50% of tumor cells were positively stained with anti-BRAF V600E (VE1) antibody, the cells with positive staining showed the signal in cytoplasm and also strong signal in nuclei, the cytoplasmic staining was non-diffuse and non-uniform. Representative images are shown in Fig. 2A-C. In the second case only small portion of tumor showed uneven, cytoplasmic staining (25% tumor cells). In addition, tumor cells also exhibited nuclear staining. Representative images are shown in Fig. 2D-F. Third case showed staining in 70% of cells, however the staining was heterogeneous and clearly nuclear along with lighter non-uniform cytoplasmic staining. Representative images are shown in Fig. 2 G-I. Fourth case showed high degree of nuclear staining, overall 90% of tumor cells showed positive staining in cytoplasm, however the strong staining was observed in nuclei with some signal in cytoplasm. This cytoplasmic staining was scattered and uneven. Representative images are shown in Fig. 2J-L. Since these four case exhibited ambiguous, non-uniform, heterogeneous and nuclear staining pattern, they were assigned as equivocal for *BRAF* V600E mutation.

In addition, there were 4 cases that were scored with stain intensity of 0.75 in all three evaluated slides in 30–90% of tumor cells. All these cases exhibited nuclear staining and non-diffuse, weak, heterogeneous cytoplasmic staining. Since the stain intensity was <1 these cases were scored as negative for *BRAF* V600E mutation.

### DNA Sequencing

Overall, out of the all 120 cases, there were 60 cases with *KRAS* mutation. Out of these 60 cases, 4 cases showed BRAF V600E stain intensity 1 in 25–90% of tumor cells. This was not expected since these *KRAS* and *BRAF* V600E are mutually exclusive mutations. These cases exhibited the staining pattern that was not consistent with the recommendations based from studies shown in Table 1 that include uniform, diffuse, cytoplasmic staining in majority of malignant cells. In addition, 4 *KRAS* mutated cases showed stain intensities 0.75 with similar nuclear/heterogeneous staining pattern. These cases were also sequenced for *BRAF* V600E mutation. All these cases were negative for *BRAF* V600E mutation by Sanger sequencing.

**Table 1:** Scoring criteria used for BRAF V600E expression in CRC by IHC

#	Author	CRC Tissues	BRAF V600E Scoring Criteria	Notes from the publications on BRAF V600E scoring/staining
1	Adackapara et al. [1]	52 cases	Scoring criteria: negative, weak, moderate, strong, (any cytoplasmic staining even a blush scored as weak)	Moderate to strong cytoplasmic staining; relatively uniform staining throughout all positive cases, non-specific nuclear staining common.
2	Affolter et al. [2].	31 cases	Scoring criteria: based on the intensity of cytoplasmic staining, percentage of tumor cells stained	Staining in the majority of BRAF mutant cases was strong and diffuse. Semiquantitative analysis of stain intensity or percentage of staining cells was not useful, because most tumor cells stained in positive cases and staining was uniformly absent in negative cases. No indeterminate cases. Heterogeneous or weak staining occurred infrequently. Cilia, nuclei of colonocytes sometimes positive.
3	Bledsoe et al. [3]	204 cases	Positive case: cytoplasmic staining, uniform to near uniform, intensity -weak to strong.	Pitfalls include signet-ring cell morphology. Dim but uniform staining should not be disregarded. Nuclear staining in normal cells. Nuclear staining occurred only in a minority of BRAF mutants, was regarded as nonspecific, and, in the absence of the cytoplasmic criteria, was taken as non-diagnostic. Nonspecific nuclear and heterogeneous, non-diffuse cytoplasmic staining of variable intensity was observed in occasional non–BRAF-mutant cases.
			Scoring criteria: negative, weak, moderate, strong. Diffuse or non-diffuse. Uniform (all malignant cells) or near uniform, heterogeneous (variable stain intensity).	
4	Capper et al. [7]	91 cases	Positive case: staining of >80% tumor cells above background	Homogenous finely granular cytoplasmic staining was seen in most cases. No single anti-BRAF V600E (VE1) antibody positive cells or positive clonal foci in otherwise negative tumors were observed.
5	Day et al. [9]	477 cases	Positive case: unequivocal cytoplasmic staining above background in the majority of invasive viable tumor cells.	Any nuclear staining, weak, cytoplasmic staining of isolated tumor cells or focal confluent staining of tumor cells in a tumor that otherwise showed no staining was scored as immune-negative.
6	Dvorak et al. [11] *	279 cases	Positive case: diffuse cytoplasmic staining of >80% tumor cells	Heterogeneous staining in 3 cases out of 238 CRC on TMA
7	Kuan et al. [19]	128 cases	Scoring criteria: 3+, 2+, 1+, 0	Scoring assessment based on stain intensity is appropriate. Weak even diffuse staining (1+) is not diagnostic and requires testing by PCR analysis.
8	Lasota et al. [20]	113 cases	Scoring criteria: negative, weak, moderately positive, strongly positive	2 KRAS cases false positive, suboptimal protocol used
9	Loes et al. [21]	99 cases	Scoring criteria: 0-no staining, 1-weak diffuse cytoplasmic staining, 2 moderate diffuse, granular cytoplasmic staining, 3- strong diffuse granular cytoplasmic staining; 0–1 negative, 2–3 positive	In positive samples the staining was homogeneous with equal intensity throughout the majority of tumor cells.
10	Rossle et al. [33]	68 cases	Scoring criteria: 0- negative, 1 weakly/moderately positive, 2 strongly positive.	Diffuse staining of variable intensity (from weak to strong) in most cases. False positive staining noted in signet ring tumor cells.
			Positive case: unequivocal cytoplasmic staining of a majority of tumor cells,	
11	Roth et al. [34]	55 cases	Positive case: Uniform diffuse cytoplasmic staining (even week) in all tumor cells	Most cases –strong, diffuse, uniform cytoplasmic staining, a few cases showed weaker but diffuse and convincingly cytoplasmic staining
12	Sinicrope et al. [39]	74 cases	Scoring criteria: 0 none, 1+ weak, 2+ medium, 3+ strong)	Homogenous staining seen in the majority of cases. Any nuclear staining or weak interspersed staining was scored as negative. Any nuclear staining or weak staining of interspersed cells was scored as negative. BRAF V600E expression was homogeneous. 100% of tumor cells stained in 75% cases, >70% cells stained in all cases.
			Positive case: at least 70% tumor cells stained	
13	Toon et al. [43]	201 cases	Positive case: diffuse strong positive staining of >75% of malignant cells	The great majority of positive cases actually demonstrated diffuse strong homogenous cytoplasmic staining in essentially all malignant cells, whereas the great majority of negative cases showed completely absent staining in all malignant cells. Patchy non-specific staining in smooth muscle cells, mucin, and colonic mucosa (with nuclear staining). Weak but diffuse staining seen occasionally in positive cases.
14	Piton et al. [28]	30 cases	Positive case: >10% tumor cells showed positive signal..	BRAF mutants – staining homogeneous, cytoplasmic, finely granular. Any nuclear staining was ignored and not scored.
15	Qui et al. [29]*	181 cases	Cytoplasmic staining	The interpretation of the results was clear. The negative and positive samples can be easily distinguished without the need of a subjective IHC scoring system based on stain intensity or percentage of positively stained cells.
16	Thiel et al. [42]	176 cases	Positive case: detectable granular cytoplasmic staining	No details
17	Hang et al. [13]	425 cases	Scoring criteria: negative (0), weak (1+), moderate (2+), strong (3+)	2 cases heterogeneous staining, 70% cells stained, 8/425 cases were called equivocal due to low stain intensity
18	Schafroth et al. [37]	33 cases	Scoring criteria: cytoplasmic staining – weak to strong	Interpretation of weak staining is challenging. About 10% of cases – weak staining, these cases should be validated by another method. Nuclear staining sometimes observed in tumor cells- considered negative. Heterogeneity – minimal.
19	Estrella et al. [12]	480 cases	Scoring criteria: 0-negative, 1- weak in <20% tumor cells, 2-moderate to strong in <20% tumor cells, 3 weak in 20–70% tumor cells, 4-moderate or strong in 20–70% tumor cells, 5 weak in >70% tumor cells, 6 moderate to strong in >70% tumor cells.	Scoring system was completely different than other studies.
			Positive case: also cases with 20–70% cells stained [8–10, 31]	
20	Vakiani et al. [45]	117 cases	Scoring criteria: Weak, moderate, strong.	In majority of the cases, positive/negative score- readily achieved. Equivocal - 4 cases, 3 cases- nuclear staining in tumor cells, 1 case focal nuclear and weak cytoplasmic, mucinous carcinoma, or signet ring.
			Positive case: >80% cell tumor staining above any background staining,	
			Equivocal case: nuclear staining with cytoplasmic staining in tumor cells.	
21	Boulagnon et al. [4]	86 cases	Positive cases: (cytoplasmic, diffuse, moderate to intense), Negative cases: no or faint cytoplasmic staining	Only 3 cases equivocal because of heterogeneous staining pattern
			Equivocal case: heterogeneous, or weak staining	
22	Sajanti et al. [36]	147 cases	Positive case: diffuse staining in the tumor cells	All BRAF mutated CRCs showed diffuse and strong staining
23	Routhier et al. [35]	25 cases	Positive case: diffuse and moderate to strong cytoplasmic staining of tumor cell.	No details.
			Negative case: isolated nuclear staining, weak staining of occasional cells or faint diffuse staining –	
24	Nolan et al. [24]	152 cases	Positive case: diffuse cytoplasmic staining of >80% tumor cells, ranging from medium to strong in strength.	This study included also equivocal category – PCR confirmatory test needed for these cases. Only 8 equivocal cases.
			Negative case: absent staining or very weak staining of similar intensity to the control normal mucosa,	
			Equivocal case: heterogeneous staining	
25	Ilie et al. [16]	489 cases	Positive case: >80% cell tumor staining, strong, distinct, homogeneous staining	Equivocal cases –additional analysis required for such cases
			Equivocal case: ambiguous, focal, moderate staining	

## Disscussion

This study evaluated 120 CRC cases with and without *KRAS* mutation to access the performance of IHC using anti-BRAF V600E (VE1) antibody for detection of *BRAF* V600E mutation. Overall, the results of these experiments demonstrates that IHC using the anti-BRAF V600E (VE1) antibody with the VENTANA OptiView DAB detection system and BenchMark ULTRA platform is a highly specific and sensitive method for the detection of *BRAF* V600E in colon cancer.

There is strong evidence from multiple studies that the IHC using anti-BRAF V600E (VE1) antibody is highly concordant with molecular tests for the *BRAF* V600E mutation. Table 2 shows a summary of 25 studies that evaluated sensitivity and specificity of IHC with anti-BRAF V600E (VE1) antibody in comparison with molecular tests using different methods (Sanger, pyroseqencing, SNapShot PCR, NGS, etc.). Altogether, 4041 patient samples were evaluated in these studies, the overall sensitivity and specificity of IHC assay using anti-BRAF V600E (VE1) antibody compared to molecular tests was 93% (934/1008) and 96% (2922/3033), respectively.

**Table 2:** Summary of immunohistochemical studies using anti-BRAF V600E (VE1) antibody compared to molecular testing

	First author	Molecular testing	Tissue source	Instrument/ Detection	*Sensitivity %*(n/N)	Specificity %(n/N)	Antibody/dilution	Antigen retrieval /antibody incubation
1	Adackapara et al. [1]	Pyrosequencing	WS	Manual/ Not specified	71% (12/17)	74% (26/35)	Spring 1:50	Citrate buffer pH 6/overnight 4 °C
2	Affolter et al. [2].	Pyrosequencing	WS	BMK ULTRA/ ultraView Amplification	100% (14/14)	100% (17/17)	Spring 1:600	Manual AR/60 min antibody
3	Bledsoe et al. [3]	Multiplex PCR, SNaPshot	TMA, WS	Leica/Bond-III	96% (57/59)	99% (143/145)	Spring 1:100	40 min EDTA buffer pH 9 / Not specified
4	Capper et al. [7]	Sanger and pyrosequencing	WS	BMK XT/ OptiView Amp	100% (11/11)	99% (79/80)	Hybridoma 1:5	64 min CC1/32 min antibody
5	Day et al. [9]	Sanger and SNaPShot	TMA, WS	BMK XT/OptiView, ultraView	100% (59/59)	100% (416/416)	Hybridoma 1:3	64 min CC1/16 min antibody
6	Dvorak et al. [11] * ++	Sanger, SNapShot, and NGS	TMA, WS	BMK XT/ OptiView	100% (86/86)	99% (191/193)	Ventana 1:50	64 min CC1/16 min antibody
7	Kuan et al. [19]	PCR	WS	BMK ULTRA/OptiView	100% (74/74)	94% (51/54)	Spring 1:200	56 min CC1/20 min antibody
8	Lasota et al. [20]**	Multiple analyses, Cobas	WS	Leica/ Bond-Max/Polymer detection	89% (24/27)	78% (64/86)	Spring 1:200	25 min Bond Epitope retrieval solution 1/30 min antibody
9	Loes et al. [21]*	Sanger and LightMix	TMA	BMK XT/OptiView	59%(13/22)	84% (63/75)	Spring 1:60	64 min CC1/16 min antibody
10	Rossle et al. [33]	Sanger and ultra-deep sequencing	WS	BMK XT/OptiView	100% (37/37)	95% (20/21)	Spring 1:200	64 min CC1/32 min antibody
11	Roth et al. [34]	Multiplex PCR	TMA, WS	Leica Bond/ Not specified	88% (28/32)	100% (23/23)	Spring 1:900	20 min Leica Bond EDTA solution pH 9/15 min antibody
12	Sinicrope et al. [39]	Multiplex PCR	WS	BMK XT/OptiView	100% (49/49)	100% (25/25)	Spring 1:45	Not specified/16 min antibody
13	Toon et al. [43]	Multiplex PCR, MS	WS	Not specified	97% (37/38)	96% (157/163)	Hybridoma/Not specified	Not specified/ Not specified
14	Piton et al. [28]	SNapShot	WS	Manual/DAKO EnVision	100% (10/10)	100% (20/20)	Spring Not specified	30 min citrate buffer pH 6/16 min antibody
15	Qui et al. [29]* ++	Sanger, RT-PCR, Cobas	WS	BMK not specified/OptiView	100% (38/38)	100% (143/143)	Ventana 1:50	64 min CC1/16 min antibody
16	Thiel et al. [42]	PCR	TMA	BMK XT/OptiView or ultraView with/ without Amp	100% (26/26)	100% (129/129)	Spring 1:2000	Not specified/ Not specified
17	Hang et al. [13]	PCR	TMA	Leica Bond-Max /Bond Polymer Refine detection	91% (21/23)	99% (397/402)	Spring 1:200	30 min Bond Epitope retrieval solution 2 /15 min antibody
18	Schafroth et al. [37]	Pyrosequencing	TMA, WT	BMK ULTRA/ OptiView	100% (18/18)	93% (14/15)	Ventana 1:50	72 min CC1/40 min antibody
19	Estrella et al. [12]	Different methods	WT	Leica Bond/Bond Polymer Refine detection	75% (106/142)	93% (315/338)	Spring 1:50	20 min TRIS-EDATA buffer pH 9/Not specified
				BMK ULTRA/ OptiView	89% (51/57)	57% (20/35)		64 min CC1/ Not specified
20	Vakiani et al. [45]	PCR, Sanger	WT	BMK XT/OptiView	94% (45/48)	96% (66/69)	Spring 1:50	32 min AR/32 min antibody
21	Boulagnon et al. [4]	RT-PCR	TMA, WT	BMK XT/ ultraView	95% (20/21) TMA 100% (21/21) WT	92% (60/65) TMA 95% (62/65) WT	Abcys EuroBio 1:50	64 min CC1/32 min antibody
22	Sajanti et al. [36]	PCR	TMA	BMK XT/ OptiView Ampl	100% (13/13)	99% (133/134)	Spring 1:2000	Not specified/ Not specified
23	Routhier et al. [35]	SNapShot	WT	Leica Bond 3/Leica Polymer Refine detection	100% (17/17)	100% (15/15)	Spring 1:100	40 min EDTA solution (Leica) /Not specified
24	Nolan et al. [24]	PCR	WT	BMK ULTRA/ ultraView Ampl	93% (14/15)	100% (59/59)	Spring Not specified	32 min CC1/32 min antibody
25	Ilie et al. [16]	Sanger, pyrosequencing	WT	BMK XT/OptiView	94.2% (32/34)	100% (276/276)	Spring 1:50	Not specified/ Not specified

*only CRC cases included

++ Ventana anti-BRAF V600E (VE1) antibody and recommended protocol used, BMK XT

**data in text and table do not match

NS not specified, AR antigen retrieval, WS whole sections, TMA tissue microarray, Ampl amplification

Out of these 25 studies, 4 publications reported lower sensitivity and/or specificity of anti-BRAF V600E antibody compared to sequencing [1, 12, 20, 21]. However, there were several problems with these studies. First, the study by Adackapara et al. analyzed 52 colorectal carcinomas with known *BRAF* mutation status determined by pyrosequencing and found that IHC had a low sensitivity (71%) and specificity (74%) for detecting *BRAF* V600E mutation compared to pyrosequencing (Table 2). They concluded that IHC using anti-BRAF V600E (VE1) antibody is not a useful surrogate for detecting *BRAF* mutation in colorectal carcinoma. However, in their experiment, manual staining with citrate buffer as antigen retrieval was employed. In our experience and the experience of others the use of acid for antigen retrieval step results in suboptimal staining that is difficult to interpret [19]. TRIS or EDTA buffers at pH = 8 proved to be retrieval agents that produced the most robust and homogenous cytoplasmic staining with anti-BRAF V600E (VE1) antibody. Similarly, Lasota et al. used in their studies Bond Epitope Retrieval Solution 1 (pH = 6) which is not an optimal solution for antigen retrieval for this assay [20].

Another important factor that may contribute to the different outcome of the studies is the interpretation of the IHC results. As multiple studies have highlighted, a proper scoring system is necessary to reduce false-positive and false-negative cases. Since *BRAF* V600E mutation is a driver mutation, a majority of tumor cells should express this mutated protein. The scoring criteria shown in Table 1 were used in the individual studies presented in Table 2 that compare IHC using anti-BRAF V600E (VE1) antibody with molecular testing for *BRAF* V600 E mutation. Overall, 14 out of 25 studies scored cases positive for *BRAF* V600E mutation when uniform or nearly uniform, diffuse staining was present in tumor cells or when the majority (≥ 75%) of tumor cells exhibited unequivocal cytoplasmic staining (Table 2). All studies that used these interpretation criteria (and appropriate protocol using antigen retrieval at alkaline pH) reached close to 100% sensitivity and specificity compared to sequencing. Additional 7 studies did not include scoring criteria in the method section, however they reported that homogeneous, diffuse staining pattern was observed in cases with confirmed *BRAF* V600E mutation. Three studies provided no details. In one study the cases were scored as positive for BRAF V600E staining when only ≥20% tumor cells showed positive signal in one study [12]. The sensitivity and specificity reported in this study was only 89% and 57% (when IHC assay on BenchMark ULTRA platform was used) and 75% and 93% (when IHC assay on Leica Bond was used). Several studies suggested that additional analysis is required for minority of equivocal cases with ambiguous, focal, heterogeneous staining (Table 2). False positive staining was noted in signet ring tumor cells [33]. Importantly, the nuclear staining was described as the most common artifact (Table 2) [22]. For example, Bledsoe et al. noted that *BRAF*-mutant cases showed homogeneous, finely granular, cytoplasmic staining with varying intensities, however, non-specific nuclear and heterogeneous non-diffuse cytoplasmic staining of variable intensity was observed in a minority of non-*BRAF* mutant cases [3]. Therefore, it was recommended by Marin et al. that “the interpretation should be made with caution in the presence of nuclear staining” [22]. Our study also suggests that the cases showing the presence of heterogeneous cytoplasmic staining with or without nuclear staining should be carefully interpreted.

Overall, the evidence from the publications presented in Table 1 and from the current study suggests that the cases should be scored as positive for *BRAF* V600E mutation if they display unequivocal, diffuse, uniform, granular, cytoplasmic staining in the majority of tumor cells at stain intensity ≥1. They should be scored as negative for *BRAF* V600E mutation if they exhibit no staining or weak, cytoplasmic, non-granular, non-uniform staining (stain intensity <1). The cases with staining of isolated tumor cells in a tumor that otherwise showed no staining should be considered negative. The cases should be considered as equivocal if they display ambiguous, heterogeneous, cytoplasmic staining with or without nuclear staining in tumor cells. If these interpretation criteria are followed the IHC with anti-BRAF V600E (VE1) antibody using recommended protocol with OptiView detection is optimal for detection of *BRAF* V600E mutation in CRC. In our study all 30 cases with *BRAF* V600E mutation showed unequivocal positive cytoplasmic staining in 85–100% tumor cells; all 30 cases with wild-type *KRAS* and *BRAF* were negative; 6.7% (4/60) cases with *KRAS* mutation showed heterogeneous, cytoplasmic/nuclear staining at stain intensity 1. However, the staining was heterogeneous and the presence of distinct nuclear staining was noted in these four cases along with cytoplasmic staining. Therefore, these cases were assigned as equivocal for *BRAF* V600E mutation. These cases were sequenced and confirmed to be negative for *BRAF* V600E mutation.

In summary, this study indicates that IHC with the anti-BRAF V600E (VE1) antibody performed on the Benchmark ULTRA automated stainer is a highly sensitive and specific detection method for determination of *BRAF* V600E mutation status in CRC. The results presented in this study are consistent with previous reports indicating that *KRAS* and *BRAF* V600E mutation are mutually exclusive. Based on our findings and consistent with other literature reports, the majority of *BRAF* V600E positive cases demonstrate a uniform or nearly uniform, diffuse staining pattern present in the majority of tumor cells. We propose that in the minority of cases with an equivocal staining pattern, additional molecular testing should be done to assess *BRAF* mutational status.
